# Supplementary material for: The global burden of chromoblastomycosis
Source: PLoS Negl Trop Dis. 2021 Aug 12;15(8):e0009611. doi: 10.1371/journal.pntd.0009611 (PMC8360387; doi:10.1371/journal.pntd.0009611)
Supplement: S1 Table — (DOCX) [file pntd.0009611.s001.docx]

| **Countries** | **Cases (n)** | **Years of publication** | **Age (years), (mean, range)** | **Male/Female**  **n (%)** | **Time from onset of symptoms to diagnosis (years)** | **Type of lesions** | | | | | | **Site of Lesion** | | | | **Density / 1.000.000 hab** |
| --- | --- | --- | --- | --- | --- | --- | --- | --- | --- | --- | --- | --- | --- | --- | --- | --- |
|  |  |  |  |  |  | **P** | **V** | **T** | **N** | **S** | **U** | **L** | **U** | **T** | **F** |  |
| **Central America** | **1,628** | **1931-2016** | **54,6 (9y-90y)** | **802 (76.7%) / 243** | **13.4 (2mo-74y)** | **39** | **162** | **6** | **46** | **1** | **7** | **472** | **288** | **42** | **13** | **-** |
| Costa Rica | 153 | 1953-2014 | NA (14y-86y) | 80 (89.9%) / 9 | 20 (ND) | 22 | 8 | NA | NA | NA | NA | 40 | 18 | 1 | 2 | 1.67 |
| Cuba | 319 | 1942-2009 | 49.8 (14y-74y) | 289 (90.6%) / 30 | 9.85 (6mo-25y) | 8 | 105 | 0 | 3 | 1 | 0 | 113 | 88 | 9 | 4 | 0.65 |
| Jamaica | 31 | 1967-1986 | 56 (20y-89y) | 26 (84%) / 5 | 8 (3mo-27y) | NA | 16 | NA | NA | NA | 6 | 20 | 9 | 0 | 1 | 0.78 |
| Honduras | 52 | 1955-1999 | 38.1 (33y-70y) | 8 (66.6%) / 4 | NA | NA | NA | NA | NA | NA | NA | 15 | 1 | 0 | 0 | 0.30 |
| Puerto Rico | 7 | 1931-1938 | 55 (40y-80y) | 7 (100%) / 0 | 14.5 (6y-28y) | 2 | 3 | 2 | 3 | 0 | 1 | 6 | 1 | 0 | 0 | 0.28 |
| Mexico | 603 | 1943-2013 | NA (9y-90y) | 380 (66.2%) / 194 | NA (2mo-74y) | 5 | 24 | 1 | 33 | 0 | 0 | 268 | 167 | 32 | 6 | 0.11 |
| Panama | 8 | 1945-2016 | 50.8 (36y-70y) | 8 (100%) / 0 | 6.12 (2.5y-25y) | 2 | 4 | 3 | 3 | 0 | 0 | 7 | 2 | 0 | 0 | 1.17 |
| Guadeloupe | 5 | 1995-2005 | 78 (58y-84y) | 4 (80%) / 1 | 22 (1y-10y) | 0 | 2 | 0 | 4 | 0 | 0 | 3 | 2 | 0 | 0 | 1.2 |
| Dominican Republic | 450 | 1966-2010 | NA | NA | NA | NA | NA | NA | NA | NA | NA | NA | NA | NA | NA | 1.57 |
| **South America** | **2,619** | **1949-2017** | **57,4 (13y-93y)** | **1,237 (87.4%) / 178** | **12.1 (1mo-50y)** | **248** | **329** | **87** | **84** | **37** | **47** | **1,021** | **301** | **54** | **47** | **-** |
| Brazil | 1,143 | 1949-2020 | 55.5 (13y-93y) | 911 (89%) / 113 | 11.2 (1mo-40y) | 214 | 295 | 78 | 81 | 30 | 44 | 807 | 173 | 35 | 40 | 0.098 |
| Venezuela | 1,167 | 1949-2005 | 56 (30y-72y) | 144 (74.6%) / 49 | 15.8 (10mo-50y) | 5 | 5 | 0 | 1 | 2 | 2 | 56 | 98 | 15 | 5 | 1.6 |
| Colombia | 167 | 1954-1974 | NA (12y-79y) | 94 (94%) / 6 | 6 (6mo-40y) | NA | NA | NA | NA | NA | NA | 85 | 14 | 0 | 0 | 0.42 |
| Ecuador | 34 | 1962-2015 | 47 (34y-58y) | 25 (100%) / 0 | NA (3y-8y) | NA | NA | NA | NA | NA | NA | 22 | 3 | 0 | 0 | 0.12 |
| Paraguay | 82 | 1935-2015 | 61.3 (21y-88y) | 46 (86.8%) / 7 | 11 (3mo-35y) | 22 | 15 | NA | NA | NA | NA | 39 | 6 | 1 | 1 | 0.31 |
| Bolivia | 3 | 2015 | 46.6 (22y-76y) | 3 (100%) / 0 | 1.5 (7mo-2y) | 2 | 1 | 0 | 1 | 0 | 0 | 2 | 1 | 0 | 0 | 0.27 |
| Peru | 7 | 1996-2017 | 59 (48y-66y) | 2 (66%) / 1 | 12.5 (8y-16y) | 3 | 0 | 0 | 0 | 0 | 0 | 0 | 2 | 1 | 0 | 0.25 |
| Argentina | 4 | 1982-1984 | 65.6 (54y-75y) | 2 (66%) / 1 | 10 (7y-15y) | 0 | 3 | 3 | 0 | 3 | 0 | 3 | 1 | 1 | 0 | 0.068 |
| French Guiana | 11 | 1962-1974 | 61.1 (46y-72y) | 9 (90%) / 1 | 11 (1y-28y) | 1 | 10 | 6 | 1 | 2 | 1 | 7 | 3 | 0 | 1 | 1.34 |
| Uruguay | 1 | 1955 | 65y | 1 (100%) | 30y | 1 | 0 | 0 | 0 | 0 | 0 | 0 | 0 | 1 | 0 | 0.3 |
| **Africa** | **1,875** | **1947-2020** | **46,1 (2y-73y)** | **1,338 (83,6%) / 263** | **9,1 (4mo-31y)** | **153** | **131** | **558** | **117** | **32** | **97** | **1,538** | **196** | **32** | **20** | **-** |
| South Africa | 156 | 1949-1992 | 53.6 (ND) | 62 (77.5%) / 18 | NA | 1 | 3 | 1 | 2 | 1 | 0 | 50 | 25 | 4 | 0 | 0.21 |
| Republic of the Congo and the Democratic Republic of the Congo | 121 | 1947-1983 | 40.9 (20y-60y) | 26 (78.8%) / 7 | 5.9 (2y-10y) | 2 | 9 | 9 | 1 | 1 | 3 | 29 | 4 | 0 | 0 | 0.3 |
| Gabon | 64 | 1970-1995 | 52 (18y-73y) | 34 (53%) / 30 | NA (8mo-31y) | 28 | 0 | 0 | 20 | 0 | 0 | 54 | 8 | 1 | 0 | 3.2 |
| Cameroon | 23 | 1947-1970 | 40.8 (40y-65y) | 3 (50%) / 3 | 15 (NA) | 2 | 4 | 1 | 1 | 0 | 2 | 2 | 0 | 0 | 0 | 0.46 |
| Angola | 7 | 1967-1970 | 52.7 (41y-60y) | 4 (100%) / 0 | 10.1 (5y-13y) | 1 | 2 | 1 | 1 | 0 | 3 | 4 | 0 | 0 | 0 | 0.23 |
| Morocco | 18 | 1989-2018 | 54.5 (40.6y-70y) | 6 (60%) / 4 | 10.7 (1y-30y) | 3 | 3 | 1 | 9 | 0 | 3 | 2 | 7 | 2 | 0 | 0.03 |
| Uganda | 34 | 1950-2019 | NA | NA | NA | NA | NA | NA | NA | NA | NA | NA | NA | NA | NA | 0.025 |
| Kenya | 33 | 1965-1973 | 38 (15y-60y) | 17 (53%) / 15 | 5 (4mo-20y) | NA | NA | NA | NA | NA | NA | 30 | 2 | 0 | 0 | 0.41 |
| Ethiopia | 14 | 1978-2010 | 33 (30y-36y) | 1 (34%) / 2 | 5 (NA) | 1 | 2 | 0 | 1 | 0 | 3 | 3 | 0 | 0 | 0 | 0.05 |
| Tanzania | 17 | 1975-1980 | 44.6 (30y-67y) | 12 (70.6%) / 5 | 8 (NA) | 0 | 1 | 3 | 6 | 0 | 8 | 14 | 2 | 1 | 0 | 0.18 |
| Senegal | 2 | 2003-2006 | 63 (58y-68y) | 2 (100%) / 0 | 11.5 (3y-20y) | 0 | 0 | 1 | 2 | 0 | 1 | 2 | 0 | 0 | 0 | 0.09 |
| Sierra Leone | 3 | 2004-2005 | NA | NA | NA | NA | NA | NA | NA | NA | NA | NA | NA | NA | NA | 0.27 |
| Tunisia | 5 | 2003-2017 | 55.8 (28y-70y) | 4 (80%) / 1 | 2.9 (1.5y-6y) | 3 | 1 | 1 | 0 | 1 | 0 | 1 | 2 | 1 | 0 | 0.12 |
| Libya | 4 | 1983-2009 | 33.2 (12y-60y) | 1 (25%) / 3 | 10 (6y-15y) | 2 | 3 | 1 | 1 | 1 | 0 | 3 | 3 | 1 | 2 | 0.17 |
| Madagascar | 1,323 | 1955-1995 | 41.4 (2y-64y) | 1,146 (86.9%) / 172 | <1y - 28y | 105 | 98 | 536 | 68 | 28 | 70 | 1121 | 138 | 20 | 17 | 3.9 |
| Zimbabwe | 35 | 1968-1975 | NA | 8 (80%) / 2 | NA | NA | NA | NA | NA | NA | NA | 14 | 1 | 0 | 0 | 0.9 |
| Nigeria | 5 | 1966-2014 | 51.9 (28y-60y) | 5 (100%) / 0 | 8.2 (3y-10y) | 2 | 3 | 0 | 2 | 0 | 2 | 5 | 1 | 1 | 1 | 0.017 |
| Reunion Island | 5 | 1958-2018 | 46.5 (40y-53y) | 1 (50%) / 1 | 25 | 1 | 1 | 1 | 2 | 0 | 1 | 2 | 0 | 0 | 0 | 2.32 |
| Comoro Island | 4 | 1990-2014 | 57.7 (43y-60y) | 4 (100%) / 0 | 5.5 (3y-15y) | 2 | 0 | 1 | 1 | 0 | 1 | 2 | 2 | 0 | 0 | 4.7 |
| Botswana | 1 | 1972 | 56 | 1 (100%) / 0 | 5 | 0 | 1 | 1 | 0 | 0 | 0 | 1 | 1 | 1 | 0 | 1.49 |
| Djibouti | 1 | 1978 | 15 | 1 (100%) / 0 | NA | NA | NA | NA | NA | NA | NA | 1 | 0 | 0 | 0 | 3.24 |
| **Asia** | **1,390** | **1930-2019** | **51.6 (7y-90y)** | **463 (71.7%) / 183** | **9.1 (20d-40y)** | **110** | **77** | **8** | **24** | **2** | **17** | **309** | **214** | **50** | **73** | **-** |
| China | 589 | 1950-2012 | 50.7 (10y-79y) | 73 (83.9%) / 14 | NA | NA | NA | NA | NA | NA | NA | 39 | 22 | 3 | 9 | 0.009 |
| Japan | 450 | 1955-2001 | 48.3 (7y-84y) | 128 (55.2%) / 104 | 8.1 (3mo-20y) | NA | 5 | NA | NA | NA | NA | 66 | 132 | 34 | 48 | 0.08 |
| Taiwan | 33 | 1995-2016 | 75.6 (29y-90y) | 23 (71.9%) / 9 | NA (2mo-20y) | 1 | 13 | NA | NA | NA | NA | NA | 1 | NA | NA | 0.09 |
| South Korea | 9 | 1990-2011 | 53.7 (37y-68y) | 3 (33.3%) / 6 | 2 (6mo-5y) | 8 | 0 | 0 | 0 | 0 | 2 | 3 | 4 | 2 | 0 | 0.009 |
| Indonesia | 13 | 1930-206 | 39 (NA) | 2 (100%) / 0 | 5 (3y-7y) | 3 | 3 | 0 | 1 | 1 | 1 | 11 | 0 | 0 | 0 | 0.0017 |
| Laos | 1 | 2011 | 72 | 1 (100%) / 0 | 10 | 1 | 1 | 1 | 0 | 0 | 0 | 1 | 0 | 0 | 0 | 0.15 |
| Vietnam | 1 | 2019 | 47 | 0 / 1 (100%) | 10 | 1 | 1 | 0 | 0 | 0 | 0 | 1 | 0 | 0 | 0 | 0.01 |
| Malaysia | 20 | 1981-2008 | 50.6 (29y-74y) | 18 (90%) / 2 | 13.8 (5mo-40y) | 0 | 16 | 0 | 0 | 0 | 3 | 19 | 1 | 0 | 0 | 0.07 |
| Philippines | 1 | 1955 | 58 | 1 (100%) / 0 | 25 | 0 | 1 | 0 | 0 | 0 | 1 | 1 | 0 | 0 | 0 | 0.04 |
| Thailand | 14 | 1994-2015 | 61.9 (45y-79y) | 9 (64.3%) / 5 | 9.3 (4mo-30y) | 6 | 2 | 0 | 2 | 0 | 0 | 3 | 4 | 0 | 1 | 0,01 |
| Nepal | 15 | 1999-2006 | 55.2 (14y-71y) | 10 (66.6%) / 5 | 20.7 (3mo-35y) | 6 | 0 | 0 | 9 | 1 | 1 | 12 | 2 | 0 | 1 | 0.065 |
| Pakistan | 2 | 2002-2019 | 17 (12y-22y) | 2 (100%) / 0 | 9 (8-10) | 2 | 0 | 0 | 1 | 0 | 2 | 1 | 1 | 1 | 2 | 0.006 |
| Sri Lanka | 71 | 1978-1993 | NA (10y-75y) | 64 (90.1%) / 7 | 5 (1mo-25y) | NA | NA | NA | NA | NA | NA | 55 | 13 | 4 | 0 | 0.29 |
| Bangladesh | 1 | 2015 | 32 | 1 (100%) / 0 | 1 | 1 | 0 | 0 | 0 | 0 | 0 | 0 | 1 | 0 | 0 | 0.006 |
| India | 169 | 1957-2016 | 43.3 (7y-81y) | 128 (81%) / 30 | 5.76 (20d-35y) | 80 | 35 | 7 | 10 | 0 | 7 | 96 | 33 | 6 | 12 | 0.003 |
| Iraq | 1 | 1973 | 70 | 1 (100%) / 0 | 3 | 1 | 0 | 0 | 1 | 0 | 0 | 1 | 0 | 0 | 0 | 0.009 |
| **Oceania** | **168** | **1947-2013** | **53.2 (19y-91y)** | **133 (88.1%) / 18** | **8.1 (1mo-30y)** | **1** | **5** | **2** | **6** | **2** | **0** | **34** | **94** | **0** | **2** | **-** |
| Australia | 158 | 1947-2013 | 54.6 (19y-91y) | 123 (87.2%) / 18 | 7.5 (1mo-30y) | 1 | 4 | 1 | 1 | 1 | 0 | 25 | 93 | 0 | 2 | 0.19 |
| New Zealand | 4 | 1992 | 40.2 (26y-65y) | 4 (100%) / 0 | 2.6 (1y-4y) | NA | NA | NA | NA | NA | NA | 3 | 1 | 0 | 0 | 1.13 |
| Solomon Islands | 1 | 2013 | 71 | 1 / 0 | 20y | 0 | 1 | 0 | 0 | 1 | 0 | 1 | 0 | 0 | 0 | 1.75 |
| New Caledonia | 5 | 1975-1989 | 47 (41y-58y) | 5 (100%) / 0 | 2y | 0 | 0 | 1 | 4 | 0 | 0 | 5 | 0 | 0 | 0 | 2.42 |
| **North America** | **25** | **1915-2018** | **54.9 (19y-79y)** | **22 (88%) / 3** | **4.8 (2mo-20y)** | **11** | **7** | **0** | **5** | **0** | **7** | **7** | **16** | **0** | **2** | **-** |
| United States | 24 | 1915-2018 | 55.82 (19-79) | 21 (87.5%) / 3 | 4.8 (2mo-20y) | 11 | 7 | 0 | 5 | 0 | 7 | 6 | 16 | 0 | 2 |  |
| Canada | 1 | 1950 | 54 | 1 | NA | NA | NA | NA | NA | NA | NA | 1 | 0 | 0 | 0 | 0.05 |
| **Europe** | **35** | **1929-2014** | **60.9 (17-85)** | **27 (77.2%) / 8** | **13.6 (3mo-31y)** | **4** | **0** | **1** | **1** | **1** | **1** | **16** | **11** | **2** | **1** | **-** |

**NA: not available; P: plaque; V: verrucous; T: tumorous; N: nodular; S: scarring; U: ulcer; L: lower limbs; U: upper limbs; T: trunk; F: face, head, and neck;**
