# Supplementary material for: The global burden of chromoblastomycosis
Source: PLoS Negl Trop Dis. 2021 Aug 12;15(8):e0009611. doi: 10.1371/journal.pntd.0009611 (PMC8360387; doi:10.1371/journal.pntd.0009611)
Supplement: Attachment — Submitted filename: PNTD-D-21-00489 Corrections requested by reviewers.docx [file pntd.0009611.s002.docx]

**June 14^th^, 2021.**

***Prof. Roderick Hay*
Guest Editor of PLOS Neglected Tropical Diseases**

***Olivier Chosidow*
Deputy Editor of PLOS Neglected Tropical Diseases**

**Manuscript number PNTD-D-21-00489**

**Dear Editors,**

First of all, thank you so much for your time, support and all helpful suggestions regarding our paper entitled “The global burden of chromoblastomycosis”.

In this revised version of our manuscript, we incorporated all reviewer's suggestions, including some adjustments to figures, references, standardization of font, and inclusion of a new figure.

Please find bellow our point-by-point answers to the reviewer’s comments.

**ANALYSES REQUIRED FOR ACCEPTANCE.**

1- **METHODS**

The editor and reviewer required no changes. We thank the reviewer for the comments.

2- **RESULTS**

**Reviewer #1.**

**Comment:** The results obtained, analysis and meta-analysis are adequate and the support of the tables and figures are adequate. Here I suggest that you add a human figure indicating (average) of the clinical locations that will make the information clearer.

**Author's reply:** Done. We incorporated a new figure (Fig3) with illustration of clinical data (distribution of lesions in different body segments and and severity of lesions) in the section “*Consolidated worldwide CBM data*”.

**Reviewer #2.**

**Comment:** Furthermore, epidemiological, clinical and mycological data, and therapeutic options were discussed accordingly. Epidemiological and mycological (distribution patterns of the genera Fonsecaea and Cladophialophora) characteristics were also implemented using clean and clear Figures (resolutions should be checked) as well a Table.

**Author's reply:** The editor and reviewer required no changes. The resolutions of the figures are in accordance with the standards requested by the website of PNTD. Click on the picture of the submitted document and it will be opened in the original version with 600dpi.We thank the reviewer for the comments.

**Reviewer #3**

**Comment:** This study provides a comprehensive review of clinical and therapeutic aspects of CBM and an estimate of the prevalence of the disease in each different area of the world. The analysis results were clearly presented. However, the Table 1 was not meeting the standard requirement of a table (better us four lines formed table). The map of each result was not very clear, especially in the agents distribution, the absulute number was not correct. For example, in China, *C. carrionii* took 60% of the pathogenic fungi, at least over 300 cases caused by this fungus.

**Author's reply:**

- The two tables, especially table 1, are in accordance with the editorial standards, based on the recommendations available in the "Tables" section (Formatting Rules and Constraints) on PLOS Neglected Tropical Diseases website. We use models of tables from other accepted papers published in the PNTD. The tables in the submitted paper have clear and organized data. The authors would like to reconsider this recommendation.

- The creation of the color scale of each map (Fig 1 and 2) was performed based on the statistical distribution of the absolute numbers through different percentiles (20,40,60,80,100).

In addition, only references with inclusion criteria described in the "Methods" section were used in this global review. The reference used with data from China was "*Lu S, Lu C, Zhang J, Hu Y, Li X, Xi L. Chromoblastomycosis in Mainland China: a systematic review of clinical features. Mycopathology. 2013 Jun; 175 (5-6): 489-95*". In this review article, etiologic agents were identified in 79 cultures and *C. carrionii* was still predominant in northern Mainland China (31/79). In the analysis of all Asia continent, *Cladophialophora* spp. represents only 9% of all etiological agents (68 strains).

3- **CONCLUSIONS**

**Reviewer #1**

**Comment:** They are adequate according to the results obtained
**Author's reply:** The editor and reviewer required no changes

**Reviewer #2**

**Comment:** The authors have addressed all relevant queries regarding CMB.

**Author's reply:** The editor and reviewer required no changes

**Reviewer #3**

**Comment:** Yes, the conclusions were supported by the data analysis and the authors discussed the distribution of CBM, the pathogenic fungi, clinical types and the treatment and prognosis. The limitations of the literatures they reviewed were mentioned. They discussed how these data could be helpful to advance our understanding of the disease burden of CBM and the public health relevance was addressed, too.
**Author's reply:** The editor and reviewer required no changes

4- **EDITORIAL AND DATA PRESENTATION MODIFICATIONS**

**Reviewer #1**

**Comment:** My suggestions are attached to the editor and the authors

**Author's reply:** As previously reported, the authors made the suggested corrections.

**Reviewer #2**

**Comment:** After reading carefully the manuscript I listed some topics for the authors consideration:

- Lines 43, 404, and 498. Malignant transformation, any specific data/type?

**Author's reply:** In the "abstract" section, the authors chose only to generically describe as "malignant transformation" due to the limited number of words. In lines 404 and 498 (redefined as 407 and 511), the authors described the types of skin neoplasms (squamous and bsal cells), but they preferred to keep the term "long history of diagnosis of CBM" as most the original reference do not provide detail between disease onset and neoplasm diagnosis, as well as on the follow up aspects.

- Line 131, dosis

**Author's reply:** Done (adjusted to Dose)

- Line 191, absolut

**Author's reply:** Done (adjusted to Absolute)

- Line 360, Exophila

**Author's reply:** Done (adjusted to *Exophiala*). The correct line is 306.

- Line 469, discrebed

**Author's reply:** Done (adjusted to Described). The correct line is 471.

- Line 480, deference

**Author's reply:** Done (adjusted to different). The correct line is 482.

- Line 483, envolvement

**Author's reply:** Done (adjusted to involvement). The correct line is 485.

- Line 469, discrebed, typos.

**Author's reply:** Done.

- Line 498, long history? Any prediction, months/years?

**Author's reply:** As related above, the authors described the types of skin neoplasms (squamous and bsal cells), but they preferred to keep the term "long history of diagnosis of CBM" as most the references do not provide a detailed description of the evolution time.

**Reviewer #3: Minor revisions are needed. Data presentation:**

**Comment:** The distribution maps, number of the pathogen.

**Author's reply:** please check response to previous comment on the same topic.

**Comment:** Table 1, the forms should be modified to four lines.

**Author's reply:** As previously reported, as described above, the authors explore justifications for table 1.

5- **SUMMARY AND GENERAL COMMENTS**

**Reviewer #1:** It is an extraordinary piece of research that could be accepted as is. It gives a clear impact of the CMB in the world and provides much more precise data. My only suggestions: The second group should be called Central America, the Caribbean and Mexico. I suggest that similar to the work of Van de Sande W (Ref 2), which was the model, they integrate a figure with the main locations, that leaves more accessible information on the clinical topography.

**Author's reply:** As previously reported, the authors made the suggested corrections (Line 196 and figure 3).

**Reviewer #2**

**Comment:** The authors have produced a nice review of chromoblastomycosis with evidence across the globe. The list of demographic, epidemiological, and clinical aspects leads it to a leading article in the field.

**Author's reply:** The editors and reviewers required no changes

**Reviewer #3:** This paper provides the most comprehensive review of the literatures published during 1914~2020, which summarized the clinical and therapeutic aspects of CBM. Through the estimate of the burden of the disease, showing CBM to be widespread, specially in Latin America, Africa and Asia. The ratio of the male to female was very high and the disease diagnosis was terribally delayed. The most infected sites and clinical patterns were analyzed. Regards to the pathogenic fungi, *Fonsecaea* spp. was the agent of more than 80% of cases. This review allows the better understanding of the epidemiological picture, and the diagnostic and therapeutic status of CBM. Though this review, we could understand the necessay of the improvement of the diagnosis and treatment of CBM and the protection of the people to aviod the infction.

**Comment:** The limitation of the review is due to the literatures were neither high quality epidemiological study nor comprehensive surveillancethe. For example, the diagnosis criteria may not meet togethor, if the phaeohyphomycosis was included or not?

**Author's reply:** All papers incorporated into comprehensive review met the main diagnostic criteria of CBM: presence of dark pigmented and thick-walled muriform cells in a biological sample. Whenever there was doubt about this finding, the paper was excluded from the analysis. This information has been included in the "Methods" section and highlighted in yellow.

**Comment:** The risk factors were not included, such as the immunocomprised patients, if the genetic susceptible patients such as CARD9 deficiency were inclu

ded?

**Author's reply:** The authors described in Lines 384-388 “The presence of immunosuppressive diseases at the time of diagnosis of CBM was reported in only 16 (0.2%) cases”, characterizing CBM as a disease of immunocompetent host. The disease has not been described in patients with “Caspase-associated recruitment domain-9” (CARD9) deficiency. The authors believe that the description of factors related to environmental exposure and clinical characteristics are more important than the attempt to explore risk factors related to disease severity in immunocompromised patients.

**Hopefully the paper may now be considered suitable for publication.**

**Yours Sincerely,**

**Dr Arnaldo Lopes Colombo, PhD**

**Laboratório Especial de Micologia, Disciplina de Infectologia, Universidade Federal de São Paulo, São Paulo-SP. Rua Botucatu, 740. CEP 04023-062 - São Paulo – Brazil**

**E-mail: arnaldolcolombo@gmail.com**

**Phone/Fax:(55)(11) 5083-0806.**

**Dr Daniel Wagner de Castro Lima Santos, MD**

**Laboratório Especial de Micologia, Disciplina de Infectologia, Universidade Federal de São Paulo, São Paulo-**

**SP. Rua Botucatu, 740. CEP 04023-062 - São Paulo – Brazil**

**E-mail: danielinfectologista@gmail.com**

**Phone/Fax:(55)(11) 5083-0806.**
